# Attenuation of Morphine Withdrawal Syndrome by Various Dosages of Curcumin in Comparison with Clonidine in Mouse: Possible Mechanism

**Published:** 2015-03

**Authors:** Majid Motaghinejad, Mohammad Yasan Bangash, Pantea Hosseini, Seyed Morteza Karimian, Ozra Motaghinejad

**Affiliations:** 1 Department of Pharmacology, School of Medicine, Iran University of Medical Sciences, Tehran, Iran;; 2 Faculty of Veterinary Medicine, University of Tehran, Tehran, Iran;; 3 Department of Physiology, School of Medicine, Tehran University of Medical Sciences, Tehran, Iran

**Keywords:** Curcumin, Morphine, Withdrawal syndrome, Visceral pain

## Abstract

**Background:**

Herbal medical compounds and their major constituent have been used in the management and treatment of opioid withdrawal syndrome and pain. This study was carried out to clarify the effect of curcumin, the major compound of turmeric, on morphine withdrawal syndrome in mouse model and its possible mechanisms of pain relieving activity by assessing in writhing test as a model of visceral pain.

**Methods:**

Due to two separate protocols (withdrawal syndrome and pain), 144 male albino mice were divided in two major groups. In withdrawal syndrome group, test effect of various dosages of *curcumin* (10, 20, and 40 mg/kg) was assessed on withdrawal signs and compared with positive and negative control and standard treatment (clonidine 0.4 mg/kg) groups. In pain groups, to determine the mechanism of pain relieving activity of *curcumin*, various dosages of *curcumin* (10, 20, and 40 mg/kg) in three separated groups, were used against acetic acid induced writhing (which is a constriction) test. The most effective dose (40 mg/kg) was used in writhing test and compared with groups pretreated with antagonist of major neurotransmitters involved in pain; and compared with group pretreated with vehicle (DMSO, 0.05%) as control.

**Results:**

*Curcumin* attenuates withdrawal syndrome in a dose dependent manner in comparison with the dependent positive control group (P<0.05). It also indicated that pretreatment with naloxone and cyproheptadine significantly attenuate antinociception effect of *curcumin* (P<0.05)*.*

**Conclusion:**

This study advocate that antinociception of *curcumin* was mediated by opioidergic and adrenergic system.

## Introduction

Herbal medical compounds are considered as major sources of new chemical medications with potential therapeutic activities. In recent years, these natural medical compounds and their flavonoids have been used in multiple mental disorders. One of these disorders is addiction.^1^ Drug dependency is a major problem all over the world. Many studies are carried out on opioidergic and non-opioidergic drugs, which can attenuate morphine withdrawal symptoms and pain suppression during withdrawal syndrome period. Most of these medications interact with dopaminergic, adrenergic, serotonergic, opioidergic, histaminergic, and purinergic systems.^2^^-^^5^

Usage of herbal medicine for the management of pain and attenuation of withdrawal symptoms has been developed in recent years, and many studies have been carried out to evaluate the effects of these medications on different methods of antinociception tests.^6^ One of the important herbal medicines is *curcumin*. *Curcumin* is a yellow pigment and one of the major constituents of turmeric rhizomes. This fraction has important medicinal and pharmacological properties. Extract of rhizomes of Curcuma longa is a major constituent of Miao-Yao-San and Jieyuwan, the traditional Chinese herbal medicines, which has been effectively used in the treatment of pain, stress, depression, drug dependency, and related disorders in China.^7^^-^^9^

Previous studies have shown that *curcumin* has antioxidant, anti-inflammatory, immunomodulatory, anticancer, and neuroprotective properties.^10^^-^^14^ Several studies indicated that curcumin has antinociceptive effect. Intraperitoneal (IP) injection of curcumin, attenuates pain perception in both visceral nociception induced by acetic acid and tail-flick test. However, the mechanisms of this antinociception activity is not clear.^15^ Previous studies indicated that naloxone (opioid receptor antagonist) or methysergide (5-HT serotonergic receptor antagonist) decrease curcumin antinociceptive activity in acetic acid induced visceral pain. Oral application of curcumin alleviates the number of abdominal construction in writhing test. These studies also showed that endogenous analgesic opioid system is involved in the curcumin-induced antinociception properties in rats.^16^^,^^17^

A study showed that single dose and chronic oral administrations of *curcumin* attenuates corneal pain in the presence and absence of morphine, in which *curcumin* enhances morphine-induced antinociception effect.^18^

The results of another study showed that chronic treatment with *curcumin*, decreases thermal hyperalgesia and increases latency phase of pain response in diabetic neuropathic pain model of mouse.^8^ Oral administration of *curcumin* decreases the latency time of pain induced by an intraplantar injection of formalin in rats.^16^

Also IP administration of *curcumin* attenuates face wiping behavior in acute and tonic phase of orofacial pain which was induced by formalin in rat.^19^ Long term administration of *curcumin* attenuates lipopolysaccharide-induced nociception in mice.^20^

Regarding these effects, the aim of this study is to determine the effect of pain relieving activity of *curcumin* by two different protocols. First, assessment of the effect of *curcumin* in the treatment and management of dependency to morphine as a standard opioid medication, and then to determine the mechanism of its antinociception by acetic acid induced writhing test.

## Materials and Methods

*Drugs*

1: *Curcumin* (Sigma–Aldrich Inc., St Louis MO, USA), 2: Acetic acid (as a nociceptive agent), 3: Naloxone (as an opioidergic receptor antagonist), 4: Cyproheptadine (as a serotonergic receptor antagonist), 5: Phentolamine (as an adrenergic receptor antagonist), 6: Chlorpheniramine (as a H1-histaminergic receptor antagonist), 7: Cimetidine (as a H2-histaminergic receptor antagonist), 8: Metoclopramide (as a dopaminergic receptor antagonist), 9: Indometacin.

All the mentioned drugs were from Sigma–Aldrich Inc. (St Louis, MO, USA) and were dissolved in dimethyl sulfoxide 5% (DMSO). We also used morphine sulphate (Temad Co. Tehran, Iran) and clonidine hydrochloride (Tolid Daru Co., Tehran, Iran). All agents were freshly prepared just before use. 

*Animals*

144 Male albino mice weighing 30-35 g were used in this study. Animals were kept in 22ºC±2 temperature and light controlled room under a 12 h light and dark cycle. Food and water were available ad libitum. The animals were allowed to adapt to the laboratory environment at least 2 hours before testing and they were used only once. All experimental procedures followed the guidelines on Ethical Standards for experiment on pain in animals and carried out according to a protocol approved by the local Animal Ethics Committee. These animals were randomly divided in two major groups: (1) Morphine withdrawal syndrome protocol and (2) Writhing test protocol. Each group was divided into subgroups (8 per subgroup) as described below.

*Morphine Withdrawal Syndrome Protocol*

48 mice were divided randomly into 6 groups:

- Group I: as “Negative control group” (independent) received normal saline for 12 days.
- Group II: as****“Positive control group” (dependent) received morphine with an increasing dosage for the first 6 days and afterwards received the highest dose of morphine for the next 6 days.
- Group III: received morphine with an increasing dosage for the first 6 days and afterwards received clonidine hydrochloride injections 0.4 mg/kg, concurrently with morphine once a day from days 6 to 12.
- Groups IV, V, and VI: received morphine for 6 days and then *curcumin* was injected with doses of 10, 20 and 40 mg/kg respectively and concurrently with morphine once a day from days 6 to 12.

*Induction of Morphine Dependency:*

Morphine dependency induced by the injection of morphine 20-45 mg/kg in 5 groups (out of 6). Morphine was injected subcutaneously with an increasing dosage manner for 6 days. This method of morphine withdrawal syndrome induction was done on the basis and similar to the previous studies.^3^^,^^21^

*Induction and Evaluation of Morphine Withdrawal Syndrome*

On the 13^th^ day, all animals in each group were injected with naloxone 3 mg/kg, and behaviors of each animal were recorded by a camera. Recorded behaviors included jumping, headshake, wet dog shake, forepaw tremor, writhing, walking sniffing, sniffing, penile liking, rearing, chewing, body grooming, face wiping, swallowing, teeth chattering. Following data processing, the count of each behavior was registered and divided by their weighing factor to obtain a single value representing the Total Withdrawal Score (TWS) (table 1). All behavioral signs were measured on the basis of previous studies.^3^^,^^21^

**Table 1:** Weighing factors (WFs) of different withdrawal signs of morphine in the mouse

**Behavior**	**WF**	**Behavior**	**WF**
1) Jumping	4	9) Body grooming	10
2) Head shake	5	10) Face wiping	10
3) Wet dog shake	5	11) Swallowing	10
4) Paw tremor	5	12) Teeth chattering	10
5) Writhing	5	13) Dysphoria	10
6) Walking sniffing	5	14) Rearing	20
7) Sniffing	5	15) Chewing	20
8) Penile licking	5	--------	--------

*Writhing Test Protocol*

This test is based on inducing nociception by intra peritoneal injection of 10 ml/kg acetic acid (0.8%). The measurement of severity of nociception is by counting the number of abdominal constrictions known as writhing. The total number of writhing was recorded by camera during 30 minutes after the injection of acetic acid. Antinociception activity of the treatment was expressed as the percentage of inhibition of abdominal constrictions in each group by the below formula: 

(Treated mean-vehicle mean×100)/Vehicle mean

This method of antinociceptive evaluation was done on the basis of previous studies.^2^^,^^23^ In addition, the onset of the first writhing was recorded as the latency time.

*Grouping of Animals*

96 male mice were randomly divided into 12 groups:

- Groups I and II: (control and indometacin treatment groups) received normal saline 0.1 ml/mice and indometacin 5 mg/kg, 15 minutes before injecting acetic acid, respectively.
- Groups III, IV, and V: (under treatment with various dosages of *curcumin*) received *curcumin* with doses of 10, 20 and 40 mg/kg, 15 minutes before injecting acetic acid, respectively.
- Groups VI, VII, VIII, IX, X, and XI: (treated with antagonist of the major neurotransmitters involved in pain). These groups were pretreated with either dopaminergic receptor antagonist (metoclopramide, 2 mg/kg), adrenergic receptor antagonist (phentolamine, 20 mg/kg), serotonergic receptor antagonist (cyproheptadine, 4 mg/kg), opioid receptor antagonist (naloxone, 2 mg/kg), histamine type 2 receptor antagonist (cimetidine, 10 mg/kg) and type 1 histamine receptor antagonist (chlorpheniramine, 10 mg/kg), respectively. 15 minutes later, all animals in these groups received dominant doses of *Curcumin* 40 mg/kg, and after a further 15 minutes, they were injected with acetic acid 0.8% 10 ml/kg for visceral pain groups.
- Group XII: as the control group, initially received vehicle (DMSO, 0.05% as 10 ml/kg) and after 15 minutes received *curcumin* 40 mg/kg and 15 minutes later were injected with acetic acid 0.8%, 10 ml/kg for visceral pain induction.

*Statistical Analysis*

All data were analyzed by SPSS statistic software for both protocols.

- *Withdrawal Syndrome Protocol Data Analysis*
The data were presented as mean±SEM. Differences between control and morphine-dependent groups were evaluated by unpaired Student’s t test. In addition, differences among groups receiving various dosages of *curcumin* were first compared by one–way analysis of variance (ANOVA) with Tukey’s post-hoc test. P value less than 0.05 was considered to indicate statistical significance.
- *Writhing Test Protocol Data Analysis:*
The data were presented as the mean±SEM. Differences between various dosages of *curcumin* and control or indometacin were analyzed separately by unpaired Student’s t test. In addition, differences between each group of pretreated with antagonists and *curcumin* dominant dose (40 mg/kg) or vehicle analyzed separately by unpaired Student’s t test. P value less than 0.05 was considered to indicate statistical significance.

## Results

*Total Withdrawal Score (TWS) Results*

**Figure 1:**
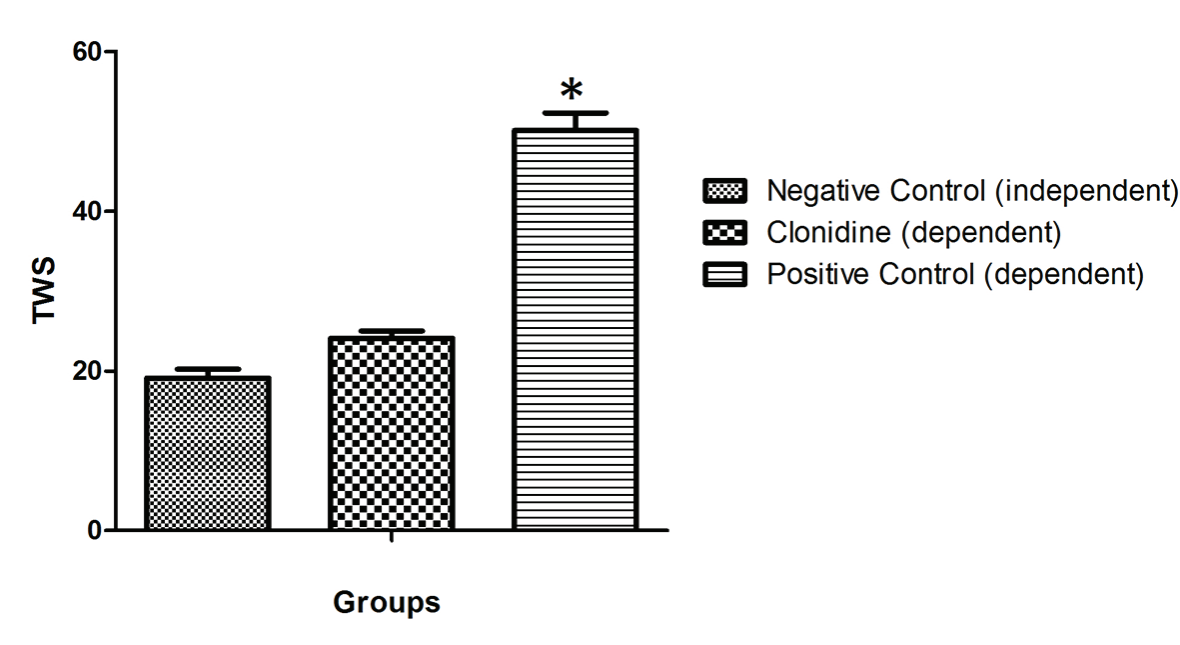
Total withdrawal score (TWS) in negative control group (independent), treatment group by clonidine (dependent) compared with positive control group (dependent). Data are mean±SEM. N=8 per group; *(P<0.05) compared with groups of negative control and treatment by clonidine.

Treatment of animals with naloxone: TWS in control morphine dependent group was 50.1±2.2 and in negative control group (with no dependency) was 19.1±1.1, which was significantly different (P≤0.05) (figure 1).

Administration of clonidine (dependent, group III) decreased the TWS to 24.1±0.9. In comparison with positive control group (dependent), this was significant but such attenuation is not statistically significant in comparison with the negative control (independent) group (P<0.05) (figure 1).

**Figure 2:**
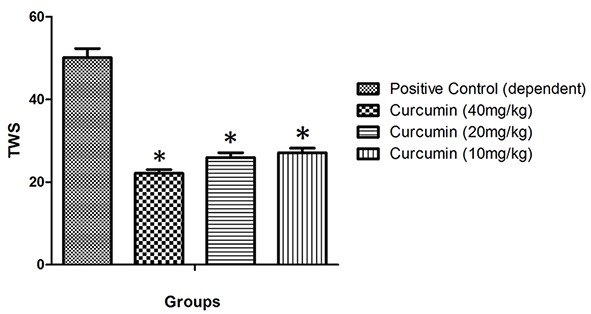
Total withdrawal score (TWS) in mice at three doses of curcumin, compared with positive control group (dependent). Data are mean±SEM. N=8 per group; *(P<0.05) compared with groups under treatment by various doses of curcumin.

Administration of *curcumin* with three doses (40, 20, and 10 mg/kg) decreased TWS to 22.1±0.9, 25.9±1.2, 27.1±1.1, respectively. These attenuations were significant in comparison with positive control (dependent) group and there was no significant difference with normal group (P<0.05) (figure 2).

*Evaluation of Antinociceptive Effects of Curcumin in Writhing Test*

The results showed that various dosages of *curcumin*****cause significant reduction in pain response in a dose-dependent manner in comparison with the control group (P<0.05). Indometacin, in comparison with the control group, significantly decreased the number of writhing (P<0.05). The percentage of inhibition of writhing response exhibited by *curcumin* extract at doses 10, 20 and 40 mg/kg were 23%, 26%, and 39% respectively. Note that, indometacin inhibited the writhing response by 74% (table 2).

**Table 2:** Effect of various doses of *Curcumin* in acetic acid-induced writhing test in mice

**Treatment**	**Dose (mg/kg IP)**	**Latency Time (sec)**	**Writhing test Inhibition P value (Mean±SEM)**	**Inhibition % P. value%**	**P value**
Control	10 (ml/kg)	337±40	63±5.1	------	------
*Curcumin*	10	389±50	48±1.7	23	0.0145 vs. control
*Curcumin*	20	455±26	46±4.6	26	0.0069 vs. control
*Curcumin*	40	611±21	38±0.4	29	0.0002 vs. control
Indometacin	5	786±66	16±3.1	74	<0.0001 vs. control

N=8 for each group

*Effect of Various Antagonists of Neurotransmitter on the Antinociceptive Action of Curcumin*

The results showed that 40 mg/kg of *curcumin*, induced a significant reduction in pain response (33 %) compared with control group (DMSO 5%) (P<0.05). The data also showed that, pretreatment with naloxone and cyproheptadine (as opioidergic) and serotonergic system antagonist, significantly reduces the antinociceptive effect of *curcumin*. Such reduction was significant in comparison with *curcumin* (40 mg/kg) group (P<0.05). Pretreatment with phentolamine (adrenergic antagonist), chlorpheniramine (H1-histaminergic antagonist), cimetidine (H2-histaminergic antagonist), and metoclopramide (dopaminergic antagonist) did not show significant change in pain response when compared with *curcumin* (40 mg/kg) group; but it showed a significant difference with control group (DMSO 5%) (P<0.05) (table 3).

**Table 3:** Effects of neurotransmitters antagonists on the antinociceptive action of* Curcumin *(40 mg/kg)

**Treatment**	**Dose ** **(mg/kg IP)**	**Latency** **Time (sec)**	**Writhing test Inhibition P value** **(Mean±SEM)**	**Inhibition % P value%**	**P value**
*Curcumin*	40	614±19	42±0.3	33	0.0002 vs.vehicle
Naloxone+*Curcumin*	2+40	30±1.6	55±1.8	12	0.0009 vs. *curcumin*
cyproheptadine+*Curcumin*	4+40	216±13	53±1	19	0.0045 vs. *curcumin*
Phentolamine+*Curcumin*	20+40	603±11	43±0.4	31	0.0003 vs. vehicle
Chlorpheniramine+*Curcumin*	10+40	605±14	44±0.6	30	0.0005 vs. vehicle
Cimetidine+*Curcumin*	10+40	600±17	40±1	36	0.0001 vs. vehicle
Metoclopramide+*Curcumin*	2+40	601±12	46±0.5	26	0.0013 vs. vehicle
Vehicle (DMSO 5%)	10 (ml/kg)	324±34	63±4.2	------	-----

N=8 for each group

## Discussion

This study showed that *curcumin* could decrease withdrawal syndrome symptoms in a dose dependent manner in morphine dependent mouse. Our data also show that *curcumin* activity in withdrawal syndrome management is mediated by potent antinociceptive effect of this compound. In visceral pain induced model, *curcumin* decreased abdominal constrictions (writhing) of mice, which were induced with acetic acid. This result showed that antinociception effect of *curcumin* was mediated by opioidergic and serotonergic system; as confirmed by the results in other studies.^24^ In order to evaluate the effect of this compound in morphine dependency, the standard protocol of withdrawal syndrome was performed. For the assessment of *curcumin* antinociception activity and possible mechanism of its effect, writhing test were studied on antagonists of six neurotransmitters that were implicated and involved in pain perception.

Many studies have been carried out on the evaluation of brain neurotransmitters. They all confirm the critical role of these peptides and amines such as dopamine, adrenaline, serotonin, histamine and opioids like peptides in pain perception and management.^5^ Additionally, they confirm the effect of these neurotransmitters in morphine withdrawal syndrome that probably anecdote of their impression in morphine and other opioids dependency. In many studies and pharmacotherapy protocols on the pain of withdrawal syndrome, medications that manage these neurotransmitters are used.^25^ In previous studies, all compounds and medications that were used for the attenuation of withdrawal syndrome were long acting opioidergic and non opioidergic drugs. These drugs can increase concentration of dopamine, adrenalin, serotonin and other neurotransmitters in synaptic space.^5^ Previous studies proved the effect of these neurotransmitters in pain management.^26^^,^^27^ In recent years, increased usage of herbal compound and traditional medicine in neuronal disorder, such as dependency and pain, exacerbated the need for identification of their mechanisms.^28^^,^^29^ Pervious study demonstrate the effect of important compounds of herbal medication on the mentioned neurotransmitter receptors that mimics their effects.^30^ *Curcumin* is one of such herbal medicines that several studies indicate that it has antinociceptive effects. It is a major component of turmeric rhizomes, which has important medicinal and pharmacological properties. Past studies have demonstrated that this fraction can modulate the pain signaling pathway in the brain.^17^^,^^31^ In this study, *curcumin* was administrated at doses of 10, 20 and 40 mg/kg and the severity of morphine dependency was measured by naloxone induced precipitated withdrawal syndrome. Our study indicates that *curcumin* as a dose dependent manner significantly attenuates the withdrawal signs in comparison with control positive (dependent) mice. In addition, clonidine at the dose of 0.4 mg/kg, as a standard treatment of morphine dependency, causes significant decrease in opioid abandonment sign and its severity. Our data confirm previous results that clonidine is effective in the management of withdrawal syndrome.^32^ Our results also indicates that *curcumin* is effective in attenuating withdrawal syndrome in a dose dependent manner. Furthermore, there is a significant difference between the severity of morphine cessation in morphine dependent without treatment and morphine dependent under treatment by various dosages of *curcumin*. This difference is more potent in the group treated with 40 mg/kg of *curcumin*. The data also suggest that, there is no statistical difference between *curcumin* treatment groups and clonidine treatment group. It is therefore concluded that *curcumin* can be used as an adjunct or substitute treatment of opioid withdrawal syndrome.

Having established the effectiveness of *curcumin* in reducing morphine withdrawal syndrome, a study was performed on the possible mechanisms involved in morphine abandonment induced pain relief in acetic acid induced abdominal constriction test. The corresponding data indicate that pretreatment with *curcumin* at doses of 10, 20, and 40 mg/kg significantly attenuate the count of abdominal constriction in comparison with control (saline treatment) group. Our data also indicated that there is a statistical difference in the count of abdominal constriction between *curcumin* treatment groups and indometacin treatment group. Current study also confirms previous researches on indometacin efficacy in the management of acetic acid induced writhing test.^33^ The data suggest that *curcumin* can be used as adjunct therapy for pain relief in combination with indometacin and other painkillers.

It is reported that *curcumin* has potential synergistic effects with NSAIDs and prostaglandin producer inhibitors in decreasing orofacial pain in rats.^19^ On the basis of this study, *curcumin* can reduce inflammation and pain mediators. It was indicated that *curcumin* has a potential capability for inhibiting the activation of inflammatory mediators such as cyclooxygenase-2 (COX-2), lipoxygenase, and inducible nitric oxide synthase products.^34^ Our data illustrated that there are statistical differences between groups under treatment with *curcumin* pretreated with naloxone and cyperoheptadine in comparison with group under treatment of *curcumin* alone. We can also conclude that pretreatment with naloxone and cyperoheptadine decreases *curcumin* antinociception activity. These findings are confirmed by the results in other studies.^16^^,^^35^ Our results also showed that there is no difference between group pretreated with phentolamine, chlorpheniramine, cimetidine and metoclopramide in comparison with the group treated with *curcumin* alone. Therefore, it suggests that *curcumin* antinociceptive activity is not mediated by adrenergic, histaminergic, and dopaminergic systems. However, the precise mechanisms and signaling pathways of curcumin antinociceptive effect remain unclear. In the present study, these doses were only used in animal models and its effective dose in human could be different. We recommend that in future studies, the exact mechanism of *curcumin* effects should be evaluated and determined by developing molecular methods and clarify its molecular and signaling pathways effect.

## Conclusion

This study demonstrated that curcumin could be useful in attenuating the adverse effect of withdrawal syndrome. Based on our findings, antinociceptive activity of *curcumin* in a mouse model of visceral pain was mediated by opioidergic and serotonergic systems that confirms the fact that *curcumin* has opoidergic like activity. Therefore, the usage of *curcumin* could be practically effective in attenuating the pain of opioid withdrawal period.
